# High-frequency voltage oscillations in cultured astrocytes

**DOI:** 10.14814/phy2.12400

**Published:** 2015-05-11

**Authors:** Wiebke Fleischer, Stephan Theiss, Johannes Slotta, Christine Holland, Alfons Schnitzler

**Affiliations:** 1 Institute of Clinical Neuroscience and Medical Psychology, Medical Faculty, Heinrich Heine UniversityDüsseldorf, Germany; 2 RESULT Medical GmbHDüsseldorf, Germany

**Keywords:** Calcium channels, extracellular recording, Glia, HFO, MEA, multi-electrode array, P2X channels, voltage oscillations

## Abstract

Because of their close interaction with neuronal physiology, astrocytes can modulate brain function in multiple ways. Here, we demonstrate a yet unknown astrocytic phenomenon: Astrocytes cultured on microelectrode arrays (MEAs) exhibited extracellular voltage fluctuations in a broad frequency spectrum (100–600 Hz) after electrical stimulation. These aperiodic high-frequency oscillations (HFOs) could last several seconds and did not spread across the MEA. The voltage-gated calcium channel antagonist cilnidipine dose-dependently decreased the power of the oscillations. While intracellular calcium was pivotal, incubation with bafilomycin A1 showed that vesicular release of transmitters played only a minor role in the emergence of HFOs. Gap junctions and volume-regulated anionic channels had just as little functional impact, which was demonstrated by the addition of carbenoxolone (100 *μ*mol/L) and NPPB (100 *μ*mol/L). Hyperpolarization with low potassium in the extracellular solution (2 mmol/L) dramatically raised oscillation power. A similar effect was seen when we added extra sodium (+50 mmol/L) or if we replaced it with NMDG^+^ (50 mmol/L). The purinergic receptor antagonist PPADS suppressed the oscillation power, while the agonist ATP (100 *μ*mol/L) had only an increasing effect when the bath solution pH was slightly lowered to pH 7.2. From these observations, we conclude that astrocytic voltage oscillations are triggered by activation of voltage-gated calcium channels and driven by a downstream influx of cations through channels that are permeable for large ions such as NMDG^+^. Most likely candidates are subtypes of pore-forming P2X channels with a low affinity for ATP.

## Introduction

Regarded as merely passive glue to stick neurons together a hundred years ago, glial cells are nowadays appreciated for their diverse and sophisticated functions within the central nervous system. Especially, astrocytes – the most abundant cell type in the mammalian CNS – gain more and more attention for maintenance but also strong interaction and modification of neuronal function. The term “tripartite synapse” was established when it became clear that the flow of information between pre- and postsynapse is modulated by astrocytes as a third partner. Astrocytes take up potassium, purge the synaptic cleft from the neurotransmitter glutamate, replenish neurotransmitter pools by providing neurons with the precursor glutamine, and affect the synaptic plasticity of neurons via diverse mechanisms (Barker and Ullian 2010).

Astrocytes are regarded as electrically passive in comparison to neurons, and it has been widely accepted that they process information mainly via changes in intracellular calcium: They react to certain stimuli with calcium oscillations that trigger a multitude of downstream signaling events. These observations merged into a concept of astrocytic calcium excitability. Many excellent reviews have been published that summarize signaling pathways of astrocytes and their interaction with neurons (Araque and Navarrete 2010; Halassa and Haydon 2010; Santello et al. 2012; Zorec et al. 2012; Ota et al. 2013).

Increased cytosolic Ca^2+^ levels in astrocytes can be triggered by electric (Hassinger et al. 1996) or mechanical stimulation, (Guthrie et al. 1999; Stout et al. 2002) by glutamate (Cornell-Bell et al. 1990) or ATP (Jeremic et al. 2001, 2001), by bradykinin (Parpura et al. 1994; Liu et al. 2009), by hyperosmolarity (Reetz et al. 1997; Pangrsic et al. 2006) or by low-extracellular Ca^2+^ (Zanotti and Charles 1997). A transient increase in intracellular Ca^2+^ can lead in return to the release of neurotransmitters like ATP, adenosine, or glutamate and of neuromodulators like taurine and d-serine. These substances are thus summarized under the term “gliotransmitters”.

A variety of possible release sites for these gliotransmitters has been discussed. There are indications that astrocytes possess an exocytotic vesicular release machinery similar to neurons, but release of transmitters was also demonstrated through hemichannels and through volume-regulated anionic channels (VRAC) after cell swelling. There is also evidence that ATP and glutamate leave the cells through membrane channels that open upon prolonged activation as large diameter pores: Some members of the ATP-gated P2X receptors are able to form pores that permit passage of molecules as large as fluorescent dyes.

Calcium signaling is not restricted to single astrocytes but covers smaller cell assemblies by propagating calcium waves. The presence of calcium-waves originating from one astrocyte to surrounding cells was first reported by Cornell-Bell et al. in 1990; where they were elicited by extracellular glutamate (Cornell-Bell et al. 1990). The observation that ATP induces Ca^2+^ influx into astrocytes was already made in the 1980s but it was Guthrie et al. 1999 who claimed that ATP serves as an extracellular messenger released from astrocytes to mediate calcium waves (Guthrie et al. 1999). These calcium waves cover a distance from 100 to 250 *μ*m and are either propagating from cell to cell via gap junctions or regenerated in each cells after extracellular stimulation with ATP (Bennett et al. 1991; Finkbeiner 1992; Bowser and Khakh 2007). Cell swelling of astrocytes in connection with brain oedema also triggers Ca^2+^ signaling, at least partly dependent on activation of purinergic receptors by ATP (Darby et al. 2003; Nase et al. 2008; Akita et al. 2011; Thrane et al. 2011).

Astrocytes participating in a Ca^2+^ wave show a distinct oscillatory pattern in intracellular Ca^2+^ with decreasing frequency (Charles et al. 1991; Pasti et al. 1997). But compared to neuronal signaling, astrocytic Ca^2+^ oscillations are very slow. Commonly used sampling frequencies used for calcium imaging are in the range from 0.5 to 10 Hz.

High-frequency voltage oscillations (250–400 Hz) have been observed in cortical and hippocampal cell cultures upon electrical stimulation on microelectrode arrays (MEAs), but were not assigned to a certain cell type (Hales et al. 2012). This represented a new application field for MEAs that were so far used to detect action potentials in dissociated neuronal or cardiomyocyte cell cultures or to measure summated postsynaptic potentials in the form of local field potentials in slices.

In this work, we show that astrocytes are the cellular source of very fast and long-lasting extracellular voltage oscillations in reaction to an electrical stimulus. Not strictly periodic, these oscillations cover a broad frequency spectrum up to 600 Hz with decreasing frequencies over time. The oscillations proved to be very susceptible to changes in the extracellular environment.

## Methods

### Ethical approval

All animal procedures were performed in accordance with the German animal care and ethics legislation and were reported to the local government authorities.

### Cell cultures

Mixed cortical cultures where prepared from newborn (P0) Wistar rats of both genders provided by the local animal laboratories. Pups were anaesthetized with isoflurane and killed by decapitation. Cortices were removed and cut into three coronal slices in phosphate-buffered saline (PBS). Cortex tissue was dissected from the middle slice which contained the striata and the anterior part of the thalamus. Cortical tissue was collected and cut into small pieces with a scalpel and then transferred into trypsin (0.05 %; 37°C for 5 min). Cells were washed several times in PBS and medium (Dulbecco's Modified Eagle's medium [DMEM] with 25 mmol/L glucose and 25 mmol/L HEPES [4-(2-hydroxyethyl)-1-piperazineethanesulfonic acid], supplemented with 10 % fetal calf serum and 25 *μ*g/mL insulin) and triturated several times with a pipette. Dissociated cells were counted and one drop of the cell suspension with a density of 1 × 10^6^ /mL was plated on poly-d-lysine- and laminin-coated microelectrode arrays (Multi Channel Systems, Reutlingen, Germany). MEAs were placed into a 5 % CO_2_ incubator with 37°C and 90 % humidity. The next day, 1.5 mL of B27-neurobasal medium with the mixture of penicillin and streptomycin (Pen/Strep), glutamine (500 *μ*mol/L), and Basic fibroblast growth factor (2 ng/mL) was applied onto the MEAs.

Cultured cortical astrocytes were also prepared from P0 Wistar rats. After a 10 day proliferation phase in T75 flasks, astrocytes had grown confluent and were detached from the flasks with trypsin (0.05 %; 37°C for 2 min) and after several washing steps plated on poly-d-lysine-coated MEAs or coverslips. Astrocytes were maintained in DMEM with high glucose and GlutaMAX (61965, Life Technologies, Carlsbad, CA) with 10 % horse serum, 1 x Pen/Strep, 1 mmol/L sodium pyruvate in an incubator with 10 % CO_2_ (37°C and 90 % humidity).

Mouse embryonic fibroblasts (MEF) were obtained from E13 embryos. Briefly, head and inner organs were removed; the remaining tissue was minced, trypsinized, and triturated with a pipette. Cells were then plated on gelatine coated flasks, cultured until confluence, and then frozen in liquid nitrogen. Upon thawing and seeding on MEAs, fibroblasts were maintained in the same nutrition medium as the astrocyte cultures. MEF were not tested for their purity with immunostainings against the marker protein vimentin.

### Immunostainings

Cultures were fixed with 4 % paraformaldehyde for 1 h at time points when parallel cultures on MEAs exhibited high-frequency oscillations. We used the following antibodies: monoclonal Anti-Glial Fibrillary Acidic Protein (GFAP) antibody produced in mouse (G3893, Sigma-Aldrich, St. Louis, MO) and Neuronal Class III *β*-Tubulin Rabbit Monoclonal Antibody (MRB-435P, Covance, Princeton, NJ). Secondary antibodies were labeled with the fluorescent dyes Alexa 488 and Cy3, respectively.

### Recording and stimulation

Extracellular recording of electric activity in dissociated cell cultures was performed with the MEA2100-System (Multi Channel Systems, Reutlingen, Germany) which provides an integrated stimulus generator and temperature control. We used microelectrode arrays (MEAs) with a square grid of 60 titanium/titanium nitride alloy (Ti/TiN) electrodes (30 *μ*m diameter, 200 *μ*m spacing) and an input impedance of <50 kΩ according to the manufacturer (Multi Channel Systems). The standard data acquisition software MC_Rack was used for visualizing and storing the extracellular potentials measured. Signals were sampled simultaneously at 25 kHz from all 60 electrodes. To attenuate stimulation artifacts, a second order high-pass filter with a cut off frequency of 100 Hz was used in the MEA2100 prior to signal amplification. As we used the same electrodes for stimulation and recording, this procedure enabled us to record a stable extracellular signal just about 50 ms after stimulus delivery. In standard measurements, we applied biphasic square wave voltage pulses (800 mV; 200 *μ*s/phase) with the negative phase first to each of the MEA electrodes (except the ground electrode). Prior to stimulation experiments, cultures were left at rest in the amplifier headstage to equilibrate for 10 min. In our experimental paradigm, each recording started with a stimulus-free baseline period of 30 sec, followed by 10 voltage pulses delivered every 20 sec, with a total recording length of 240 sec.

Recordings were made at 37°C in buffered salt solution, containing 100 mmol/L NaCl, 10 mmol/L glucose, 10 mmol/L HEPES hemisodium salt, 5 mmol/L KCl, 500 *μ*mol/L MgCl_2_, and 1 mmol/L CaCl_2_ (unless otherwise indicated). The rather low osmolarity of the buffer was initially chosen to resemble that of the cell culture medium for neurons (Neurobasal Medium). Different pH values were adjusted by adding several microliters of 1 mol/L NaOH or HCl, respectively, to the recording solution.

Cell culture ingredients were purchased from Life Technologies and all drugs from Sigma-Aldrich, except (3*S*)-3-[[3-[[4-(Trifluoromethyl)benzoyl]amino]phenyl]methoxy]-l-aspartic acid (TFB-TBOA) (Tocris Bioscience, Bristol, UK), gabazine, D-(-)-2-Amino-5-phosphonopentanoic acid (D-APV), and 6-cyano-7-nitroquinoxaline-2,3-dione, D-APV: D-(-)-2-Amino-5-phosphonopentanoic acid (CNQX) (Abcam, Cambridge, UK).

### Data analysis

Spikes and bursts were detected by custom-built software (RESULT Medical GmbH, Düsseldorf, Germany). The threshold for spike detection was set to 6.2 standard deviations of the average noise amplitude for each electrode. The actual temporal clustering of spikes in each channel was compared with a Poisson process of independently occurring spikes at the same firing rate. If the Poisson probability of an observed cluster with *n* > 3 spikes within a time interval Δt was below 0.005, this cluster was considered as burst.

**Figure 1:**
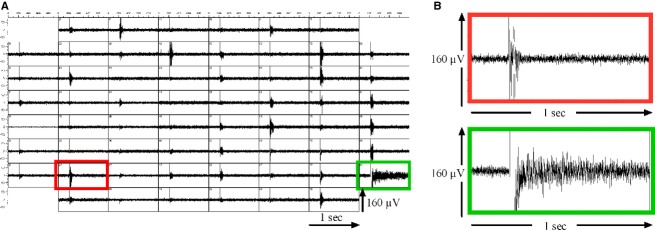
Mixed cortical cell cultures with neurons and astrocytes on MEAs exhibit spike bursts and high-frequency voltage-oscillations (HFOs). (A) A single rectangular voltage stimulus (800 mV, 200 *μ*s/phase) was applied to one MEA electrode (green frame) and triggered network bursts of action potentials detected on most other MEA electrodes. (B) An exemplary recording of one network burst (red frame) is shown at higher temporal resolution. The electrode that was used for stimulation showed long-lasting high-frequency oscillations (HFOs). These HFOs were considerably longer but less clearly defined in the time domain than spike bursts (B, green frame).

For the analysis of oscillations, continuous raw data and trigger time stamps of the recordings were imported from Multichannel Systems MCD files into MATLAB using the 64 bit Neuroshare library (version 3.7b). Individual electrodes were selected for analysis based on a clear response to stimuli in form of prolonged increased signal amplitude and controlled by visual inspection (Fig.1B, lower panel).

In order to accurately retain the temporal relation between signal and stimuli, data were subsequently downsampled to 2500 Hz after using a zero-delay linear phase finite impulse response low-pass filter of order 400 with 1125 Hz cut-off frequency. The actual time-frequency analysis of oscillatory activity was performed using the *ft_freqanalysis* function of the Field-trip toolbox (version 20130512; Oostenveld et al. 2011). To suppress stimulus artifacts, the raw signal was set to zero during 50 ms after each stimulus prior to spectral analysis. Each recording contained responses to ten stimuli occurring at 30, 50, …, 210 sec.

The time-frequency representation of the signal after each stimulus was calculated by the convolution with wavelets for time points every 50 ms and at 300 equidistant frequencies from 2 to 600 Hz. The 2 Hz frequency interval required a corresponding 0.5 sec-wide time window for the spectral power estimation. A power value calculated at a given time thus contained signal information of the surrounding 0.5 sec. Spectral analysis used a sliding temporal Hanning window centered at each time of interest and power spectra were obtained separately for each stimulus trial.

The 30 sec long baseline period was split into six nonoverlapping *pseudo*trials of 5 sec each, starting at 0, 5, …, 25 sec. Baseline power spectra were estimated in the same way as for the actual stimulus trials, and were subsequently averaged over the six *pseudo*trials and over time, yielding only frequency-dependent baseline power spectra.

Finally, time- and frequency-dependent power spectra obtained from *ft_freqanalysis* were averaged over all ten stimulus trials, and the corresponding time-independent *pseudo*trial averaged baseline power spectra were subtracted from these averaged spectra. The resulting “power over baseline” spectra were plotted as color coded maps over time and frequency.

To calculate the duration of an oscillation, spectral power was summed over all frequencies from 2 to 600 Hz, and a double exponential decay function was fitted to these *total power over baseline* data which are only time-dependent. The duration was then calculated as the time elapsed until the function fitted to the power had dropped to *exp*(-4) ≈ 1.8 % of its amplitude over background noise, which was typically at least two orders of magnitude smaller.

In order to quantify high-frequency oscillations (HFOs) by a single power estimator, we defined the *power of a HFO* as the sum of the trial-averaged *total power over baseline* for all points in time between zero and the duration of the oscillation.

In order to locate HFOs on the frequency axis, we also calculated the power-weighted median frequency in the first 0.5 sec where the oscillations were most pronounced. We used the median frequency instead of the mean, since the power distribution was heavily right-tailed and skewed towards high frequencies.

### Statistics

For all drug-related experiments, only those MEA electrodes were chosen for evaluation that showed a strong response to the applied electrical stimulus under control conditions (HFO amplitude > 3× root mean square noise), clearly distinct from the average noise amplitude. This assessment was performed by visual inspection, with no regard to the response these specific electrodes showed after pharmacological treatment. In 3.3, we compared stimulus-induced HFOs of astrocyte cultures with those of fibroblast cultures or MEAs in nutrition medium but without any attached cells. Here, we averaged power over all stimulated electrodes irrespective of HFO amplitude.

Because HFO power distribution was not normally distributed (see Fig.3), we presented the data graphically as median with upper and lower quartiles. Statistical significance in pharmacological experiments was calculated with the Wilcoxon-matched-pairs signed rank test over individual electrodes from the utilized MEAs, before and after treatment, resp. In one case where we compared two substance effects with each other [Na^+^- vs. NMDG^+^-treatment], we used the Mann–Whitney test because these observations were not paired events.

For better comprehensibility absolute HFO power was normalized to both the median value under control conditions and the mean of control. Generally, substance effects in the result section are presented as “normalized median power over baseline in %; normalized mean ± SD; *P* value of the Wilcoxon matched-pairs signed rank test; number of evaluated electrodes”.

## Results

### Mixed cortical cell cultures exhibit high-frequency voltage oscillations

Our recordings in cortical cells cultured on microelectrode arrays (MEAs) confirmed the data presented by Hales et al. (2012): Electrical stimulation evoked voltage oscillations in a broad and continuous frequency spectrum up to 600 Hz. The amplitudes of these oscillations were comparable to extracellularly measured neuronal action potentials. Considerably, fewer electrodes showed these oscillations than spontaneous bursts of action potentials. However, we could not assign certain electrodes to one of the phenomena: Some electrodes showed oscillations but no bursts, others had bursts but no oscillations and a third group showed oscillations that were superimposed by spike bursts. A stimulus applied to one specific electrode caused a network burst on all electrodes, but HFOs were restricted to the electrode that had been stimulated (Fig.1)

The duration and amplitude of these oscillations clearly depended on the power of the stimulus: oscillations grew stronger when either stimulus duration or amplitude was increased. When the stimulus was gradually reduced, oscillations became weaker until they were undistinguishable from the noise level. No threshold stimulus for the HFOs could be determined.

In accordance with Hales et al. (2012), we saw that oscillations developed over cultivation time, which might imply that single cells are not able to generate HFOs and maturing interconnections between cells are necessary. Interestingly, we observed synchronous network bursts covering the whole MEA some time before we were able to elicit HFOs on selected electrodes. On the other hand, oscillations persisted when spike activity started to diminish due to neuronal death. HFOs also survived during treatment with TTX and the synaptic blockers D-APV (20 *μ*mol/L), CNQX (10 *μ*mol/L) and gabazine (10 *μ*mol/L) that blocked all spike activity and fast synaptic transmission. All these observations raised the suspicion that the cellular source of HFOs might not be neurons but a different cell type that is tightly connected with neuronal function, probably astrocytes. All further experiments within this work were therefore performed in astrocyte-enriched cultures with a negligible amount of neurons.

### High-frequency oscillations are generated by astrocytes

**Figure 2:**
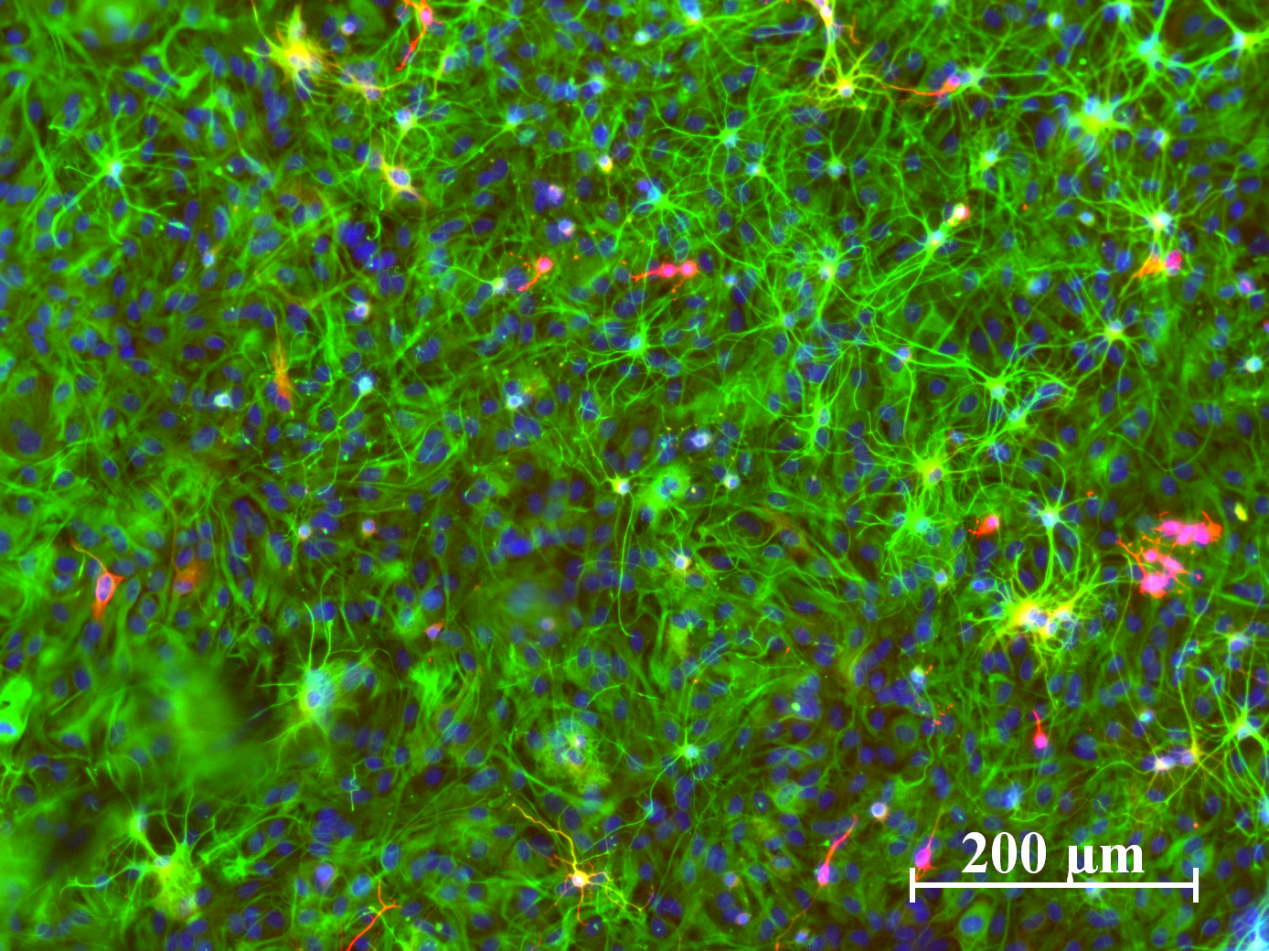
Only very few neurons in astrocyte cultures. Immunostainings of our astrocyte cultures showed a high ratio of GFAP+ cells and only a few interspersed neurons in red (*β*-III-tubulin). Cell nuclei were stained with DAPI (blue).

In such astrocyte-enriched cultures, a small amount of neurons usually survives the purification process that is performed to isolate astrocytes. Thus, astrocyte cultures are rarely 100 % “pure”. But immunostainings with the neuronal marker protein *β*-tubulin III confirmed that the astrocyte cultures used for our studies showed only a negligible amount of “contaminating” neurons (Fig.2) but a dense layer of GFAP (glial fibrillary acidic protein)-positive cells. GFAP is highly upregulated in astrocytes in culture. In addition, we never observed any spontaneous spike activity in cultured astrocytes on MEA. These observations make it highly unlikely that neurons are the cellular source of the observed voltage oscillations.

**Figure 3:**
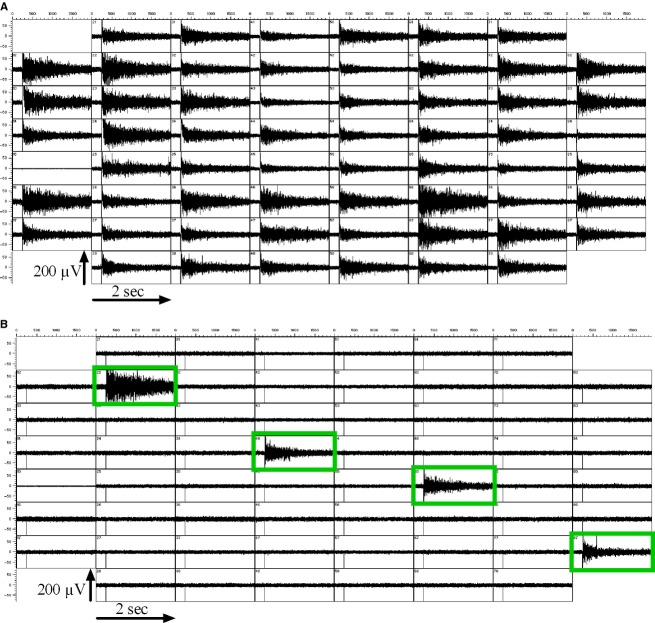
Astrocytic HFOs are locally restricted to the source of the stimulus. (A) When a stimulus was applied to the astrocyte culture via every MEA electrode (except ground), virtually all electrodes showed HFOs with varying amplitudes and durations in response. (B) When the stimulus was applied to a subset of electrodes (green frames), only cells in close proximity of the stimulated electrodes responded with HFOs. This indicates that (1) the stimulus was not passively conducted through the recording solution, and that – unlike in neuronal cultures – (2) there was no active transfer of the signal over longer distances within the cellular layer. The MEAs we used in this study featured 200 *μ*m distance between neighboring electrodes.

When biphasic voltage pulses were administered through all MEA electrodes (the grounding electrode excluded), nearly every electrode that was covered with cells exhibited high-frequency oscillations (HFOs) in return (Fig.3). Accordingly, we conclude that astrocytes generated the electrically triggered HFOs that had been observed in cortical and hippocampal cell cultures before.

In contrast to Hales et al. (2012), our power spectral analysis of the oscillations revealed no distinct frequency band. We used a hardware high-pass filter (>100 Hz) in our measurements and saw a rather wide and smooth frequency distribution with power decreasing at higher frequencies. Thus, the oscillations were rather aperiodic, and oscillation power correlated with the oscillation duration.

**Figure 4:**
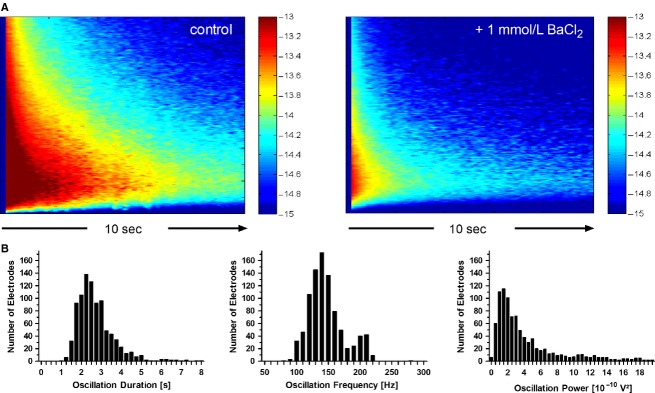
High-frequency voltage oscillations (HFOs) in cultured astrocytes after electrical stimulation. (A) shows two color-coded plots of spectral power averaged over 42 electrodes and ten stimulus trials each, obtained from one experiment on a single MEA. The left panel shows the HFO spectrum under control conditions, while the corresponding spectrum under the inhibiting effect of 1 mmol/L BaCl_2_ is presented in the right panel. Time is plotted on the horizontal axis (range 0–10 sec) and frequency on the vertical axis (0–600 Hz). The logarithm (base 10) of the time- and frequency-dependent power over baseline is color-coded in the range 10^−15^ to 10^−13^. The average HFO in the control recording was 9.2 sec long, while the 1 mmol/L BaCl-recording lasted for 3.1 sec, until its total power over baseline had dropped to 1.8 %. (B) shows the distribution of HFO duration, median oscillation frequency and power for all 905 electrodes analyzed in 53 control recordings with standard bath solution. The bimodal structure of the median oscillation frequency distribution indicates the presence of higher oscillation frequencies (~200 Hz) on a subset of electrodes. Frequencies below 100 Hz were cut off due to the imposed hardware high pass filter.

In 53 independent control recordings containing a total of 905 electrodes with HFOs, the median duration was 2.4 s (mean ± SD: 2.7 ± 1.3 sec), and the median oscillation frequency was 142 ± 28 Hz. HFO power summed for all points in time until total power over baseline had dropped to 1.8 % was 2.9 10^−10^ V² (mean ± SD: 4.4 ± 4.0 10^−10^ V²; Fig.4).

The measured HFOs were constrained to a small area around the stimulated area. Electrodes could not detect voltage oscillations when a neighboring electrode in a distance from 200 *μ*m was used for the stimulation. Oscillations were only detected by the same electrode that was used for the stimulation (Fig.3).

### High-frequency oscillations are not electrical artifacts

We performed several control experiments to rule out the possibility that the observed HFOs were merely electrical artifacts caused by amplifier electronics, electrodes, or medium.

Five MEAs with nutrition medium but without any attached cells were stored in the incubator for 3 weeks and regularly checked for HFOs. At no time point and on no electrode could an electrical stimulus induce visible voltage perturbations that exceeded the immediate but short-lived stimulus artifact (> 30 ms). In these experiments, the stimulus trigger-averaged signal power summed up in a 9 sec time window represents the combined physicochemical and electrical noise of the ionic solution, Ti/TiN electrodes and MEA amplifier.

To test for cell-type specificity of HFOs we seeded mouse embryonic fibroblasts (MEF) on eleven MEAs – a cell type usually used as feeder layer for embryonic stem cells. The fibroblasts were seeded in high density to ensure a closed cell layer across the electrode array, cultured for 3 weeks, and regularly checked for the presence of HFOs after electric stimulation. Small amplitude HFOs could be observed on few electrodes of some MEAs. As a positive control we seeded astrocytes on ten MEAs. HFO responses to electric stimuli were clearly visible only 3 days after seeding.

**Figure 5:**
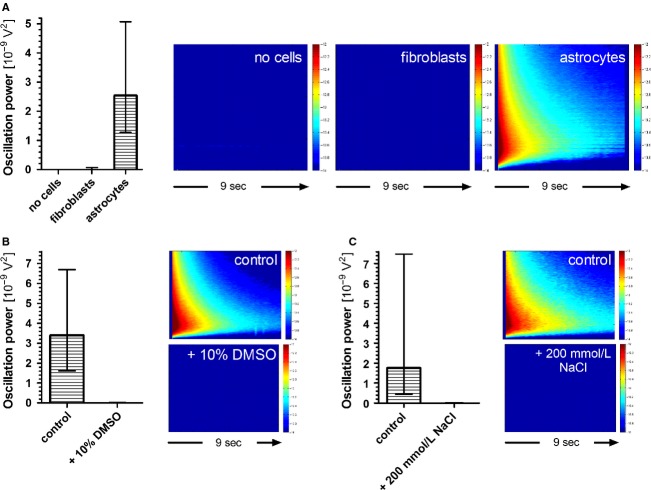
High-frequency oscillations are not electrical artifacts but generated by vital astrocytes. (A) Ten single rectangular voltage stimuli (800 mV, 200 *μ*s/phase) were applied to all 59 recording electrodes of the MEA. Signal power was summed over a time window of 9 sec after the stimulus and averaged over all 10 stimuli. The left-hand graph depicts median power and interquartile range of the voltage oscillations elicited on all stimulated electrodes. The combined physicochemical and electronic noise of the ionic solution, Ti/TiN electrodes and amplifier was determined with MEAs that were covered with nutrition medium but without any attached cells. Only very slight oscillations were observed when we stimulated mouse embryonic fibroblasts cultured on MEAs. Stimulation of astrocytes at 3 div caused strong voltage oscillations on many electrodes that exceeded the noise level (“no cells”) nearly 5000 times. Average power spectra of the respective measurements are given on the right hand side. The logarithm (base 10) of the time- and frequency-dependent power over baseline is color-coded in the range 10^−14^ to 10^−12^ V^2^. The horizontal time axis covers 9 sec after the stimulus. Frequency (0–600 Hz) is plotted on the vertical axis. (B) Astrocyte cultures were recorded before and 2 h after the addition of 10% DMSO – a concentration that is supposed to kill cells. The median power of the stimulus-induced oscillations decreased dramatically, presumably to noise level. The respective power spectra in a 9 sec window after the stimulus are depicted on the right hand side of the panel (0–600 Hz; 10^−14^ to 10^−12^ V^2^). (C) Astrocytes were exposed to hyperosmotic shock by transferring them from recording solution with 100 mmol/L NaCl to 300 mmol/L NaCl. Recordings were performed before and 2 h after the presumably deadly osmotic shock. No HFOs could be elicited after the treatment. Power spectra on the right hand side clearly demonstrate the collapse in oscillation power after the death of the astrocytes (0–600 Hz; 10^−14^ to 10^−12^ V^2^).

For these three conditions – medium, MEF, astrocytes – HFO power for each stimulated electrode (59 per MEA) was summed up over a time period of 9 sec after the stimulus. The median stimulus-averaged power of responses was 5.3 × 10^−13^ V² for nutrition medium without cells (531 electrodes; 9 MEAs), 1.4 × 10^−12^ V² for MEF cultures (1298 electrodes, 11 MEAs at two points in time) and 2.5 × 10^−9^ V² for astrocyte cultures (590 electrodes, 10 MEAs). Thus, the power of HFOs generated by astrocytes was 4800 times higher than the physicochemical noise (recordings without cells) and 1800 times higher than voltage oscillations generated by fibroblasts (Fig.5A).

In a next set of control experiments, we measured HFO responses of astrocyte cultures before and after treatments that should kill the cells but leave an intact layer of biological material covering the MEA electrodes. In a first approach, we added 10% of Dimethyl sulfoxide (DMSO) to the nutrition medium. HFOs were recorded before and 2 h after addition of DMSO. In a second approach, we induced a hyperosmotic shock to harm astrocyte integrity: Control recordings of HFOs were performed in standard recording solution with 100 mmol/L NaCl. We then applied a recording solution with 300 mmol/L NaCl to the cells. HFOs were measured again after 2 h of osmotic shock.

For the analysis, we preselected electrodes that presented a high signal-to-noise ratio under control conditions. No HFOs were visible after both treatments. DMSO addition caused a decrease in HFO power from a median value of 3.4 × 10^−9^ to 2.7 × 10^−12^ V^2^, i.e., 0.08% of the initial value (178 electrodes, 4 MEAs; Fig.5B). HFOs did not reappear after washout indicating that cells were actually dead and loss of HFO power was not due to the physicochemical properties of DMSO. Hyperosmotic shock reduced HFO power to only 0.02% of initial power, from a median value of 1.9 × 10^−9^ to 2.7 × 10^−13^ V^2^ (109 electrodes, 4 MEAs; Fig.5C). These experiments clearly show that HFOs were not electrical artifacts but are generated by living cells, especially by astrocytes.

### Oscillations are triggered by voltage-dependent calcium channels

**Figure 6:**
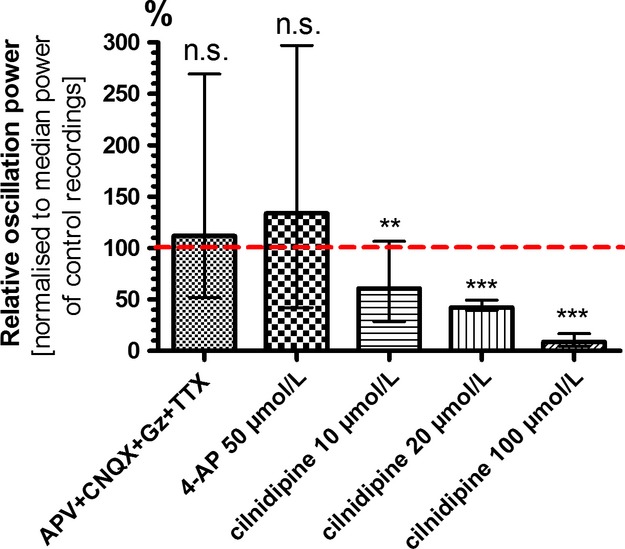
High-frequency oscillations depend on activation of voltage-gated calcium channels. Combined application of TTX (1 *μ*mol/L) with antagonists of ionotropic glutamate receptors (D-APV 20 *μ*mol/L, CNQX 10 *μ*mol/L) and GABA_A_ receptors (gabazine, 10 *μ*mol/L) showed that HFO power was insensitive to blockers of voltage-gated sodium channels and synaptic blockers. Blockade of voltage-gated potassium channels with 4-AP (50 *μ*mol/L) had just as little effect. The voltage-gated calcium channel antagonist cilnidipine inhibited astrocytic HFO power in a dose-dependent manner. These properties point to L- and/or N-type calcium channels as cellular transducers of the electrical stimulus. Data are depicted as normalized medians with inner and outer quartils (**P < 0.01; ***P < 0.001; n.s., not significant).

As electrical stimulation reliably caused HFOs in astrocyte cultures, we next investigated the mechanism of voltage stimulus sensing in these cells. In a first attempt we used a cocktail of blockers against voltage-gated sodium channels, ionotropic glutamate receptors and GABA_A_-receptors. This combination of tetrodotoxin (1 *μ*mol/L), CNQX (10 *μ*mol/L), D-APV (20 *μ*mol/L), and gabazine (10 *μ*mol/L) did not block the emergence of HFOs (112 %; 118 ± 22 %; *P* = 0.15; *n* = 28). This result precludes voltage-gated Na^+^ channels from being mediators of the electric stimulus. Parallel experiments with neuronal cultures proved the efficacy of the substance mixture. No spontaneous spike activity was seen after exposure to the mix. Incubation with the voltage-gated potassium channel antagonist 4-aminopyridine (4-AP; 50 *μ*mol/L) neither blocked HFOs nor did it alter their characteristics (134 %, 110 ± 18 %; *P* = 0.22; *n* = 35; Fig.6).

When voltage-gated calcium channels were blocked with cilnidipine (1 – 100 *μ*mol/L; Fig.6), we saw a dose-dependent decrease in the power of HFOs (10 *μ*mol/L: 61 %; 73 ± 10%, *P* = 0.0021; *n* = 66) (20 *μ*mol/L: 42 %; 38 ± 1 %, *P* ≤ 0.0001; *n* = 66), (100 *μ*mol/L: 9 %; 10 ± 1 %, *P* ≤ 0.0001; *n* = 66). Thus, we conclude that activation of voltage-gated calcium channels and subsequent influx of Ca^2+^ are relevant mediators for the development of HFOs.

### Oscillations are calcium dependent

**Figure 7:**
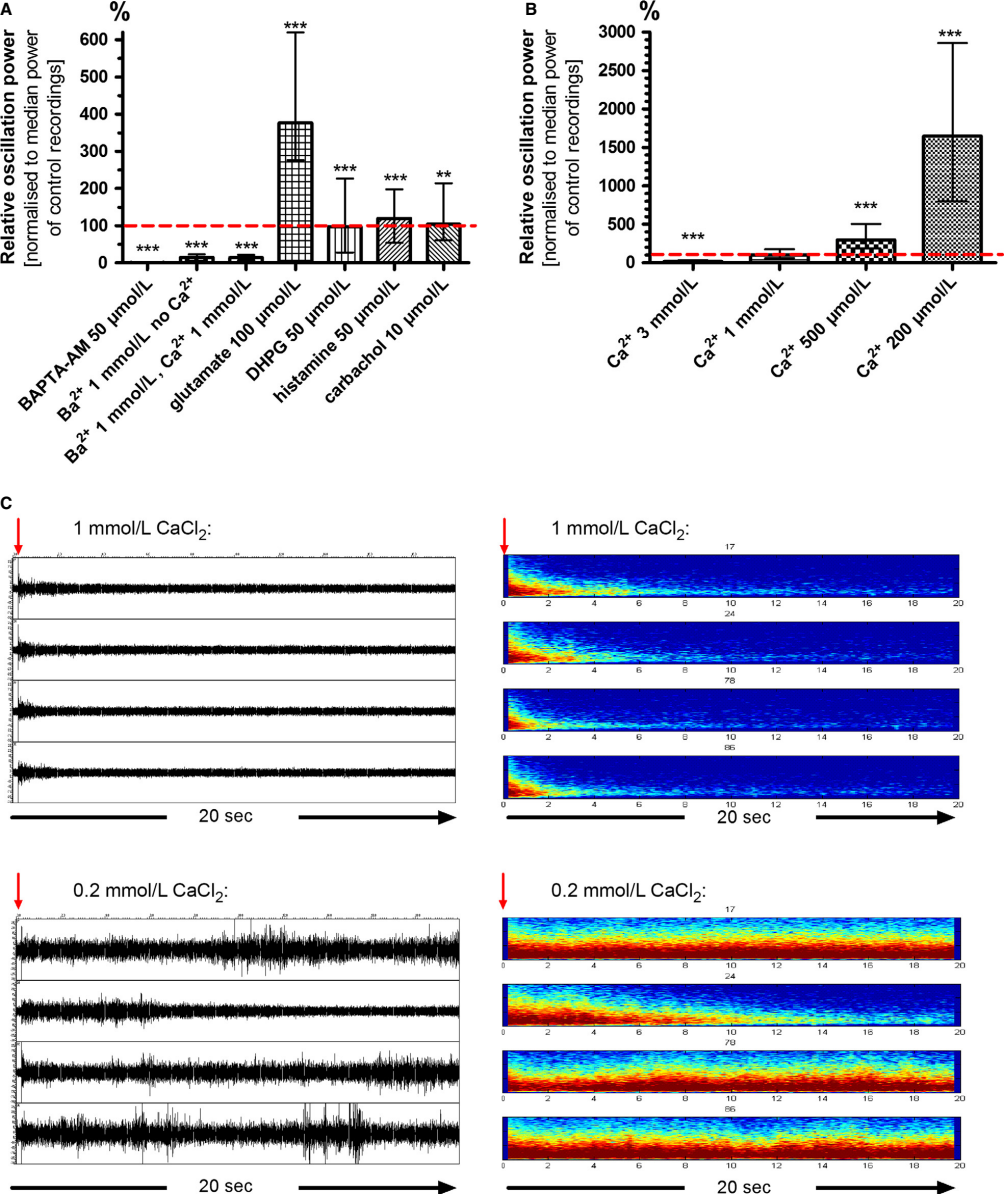
High-frequency oscillations are strongly dependent on calcium. (A) HFOs completely vanished when astrocytes were pre-incubated with the membrane-permeable Ca^2+^-chelator BAPTA-AM (50 *μ*mol/L). Substitution of extracellular Ca^2+^ with Ba^2+^ (1 mmol/L) strongly reduced HFO power. Apparently, influx of Ca^2+^ is mandatory to elicit HFOs. HFOs were as strongly suppressed when equimolar amounts of Ca^2+^ and Ba^2+^ were present in the extracellular solution. Ba^2+^ seems to be more preferably transferred across the membrane when both ions compete for it. Glutamate (100 *μ*mol/L) intensified HFO power more strongly than DHPG, an agonist of group I metabotropic glutamate receptors. Histamine (50 *μ*mol/L) and the muscarinic agonist carbachol (10 *μ*mol/L) only slightly increased HFO power (**P < 0.01; ***P < 0.001). (B) Lowering the extracellular calcium concentration resulted in a dramatic increase in HFO power: 500 *μ*mol/L tripled the power and 200 *μ*mol/L actually increased power 17-fold compared to standard conditions with 1 mmol/L CaCl_2_. The opposite effect was seen with higher calcium concentrations. Data in A and B is depicted as normalized medians with inner and outer quartils. (C) The left hand side depicts signals of four selected MEA electrodes over a time period of 20 sec after electrical stimulation (red arrow) under control conditions (1 mmol/L CaCl_2_, upper panel) and under extracellular calcium deficiency (200 *μ*mol/L CaCl_2_, lower panel). The right hand side shows the power spectra of the same MEA electrodes averaged over ten stimuli applied at 20 sec intervals. HFOs under control conditions were small in amplitude and lasted approximately 2 sec. With only 200 *μ*mol/L Ca^2+^, the HFOs elicited by the voltage pulse on the same electrodes persisted even after the inter-stimulus interval of 20 sec. Note that HFOs on most electrodes showed no gradual decrease in amplitude but rather spontaneous rekindling. Power was maximal at frequencies below 200 Hz.

In order to investigate the role of intracellular calcium for HFOs, we incubated astrocyte cultures with the membrane-permeable chelator 1,2-Bis(2-aminophenoxy)ethane-N,N,N′,N′-tetraacetic acid tetrakis(acetoxymethyl ester) (BAPTA-AM). BAPTA-AM moves into the cells and “captures” intracellular Ca^2+^ ions thereby suppressing any downstream-signaling events triggered by this second messenger. To rule out that all binding sites of the chelator were already fully occupied before it entered the cell we incubated the cultures with Ca^2+^-free solution and added 50 *μ*mol/L BAPTA-AM. After 1 h incubation and washout we were not able to elicit any HFOs, neither in Ca^2+^-free nor in standard bath solution, which indicates a strong dependency of HFOs on intracellular Ca^2+^ (HFO power: 1 %; 0.8 ± 0.1%; *P* ≤ 0.0001; *n* = 29). However, as all cultures that underwent this treatment were dead on the following day we cannot be completely sure whether the complete suppression of HFOs is a proof of its calcium dependency or simply a symptom of dying astrocytes (Fig.7A).

To get more convincing evidence for an intracellular Ca^2+^-dependent signaling cascade we used BaCl_2_ combined with or instead of CaCl_2_ in the extracellular solution. Under both conditions, HFOs were strongly suppressed: The power of the oscillations decreased to a median value of 14 % (14 ± 3 %; *P* ≤ 0.0001; *n* = 29) when equimolar amounts of CaCl_2_ and BaCl_2_ were used (1 mmol/L each) and to 15 % (12 ± 1 %; *P* ≤ 0.0001; *n* = 74) when only Ba^2+^ but no Ca^2+^ was added to the bath solution (Fig.7A). This inhibiting effect of BaCl_2_ is probably due to the “Ca^2+^-imitating” qualities of barium: Both are divalent cations from the group of alkaline earth metals and are able to enter cells via Ca^2+^ channels. But Ba^2+^ does not act as a secondary messenger-like Ca^2+^ does.

From these experiments, we conclude that HFOs can only be generated, if Ca^2+^ enters the cells and mediates internal signaling routes. Voltage-gated Ca^2+^ channels that open only after strong depolarization (high-threshold or high-voltage-activated Ca^2+^ channels) show a higher permeability for Ba^2+^ than for Ca^2+^ (Yatani et al. 1987). The strong inhibition in experiments where Ba^2+^ did not replace Ca^2+^ but was added in equimolar amounts (1 mmol/L) to the recording solution suggests that Ca^2+^ influx via such high-threshold channels is essential for HFO generation. In agreement with these results, the calcium-channel antagonist cilnidipine blocks both L- and N-subtypes of high-voltage-gated calcium channels.

To test if a rise in intracellular Ca^2+^ was sufficient to elicit HFOs we applied several agonists to the cultures that are known to raise Ca^2+^ within the cell via activation of G protein-coupled receptors: (S)-3,5-Dihydroxyphenylglycine (DHPG) is an agonist of group I metabotropic glutamate receptors, histamine increases intracellular Ca^2+^ via activation of H1 receptors and carbachol (carbamoylcholine chloride) is an agonist for muscarinic acetylcholine receptors. None of the tested substances caused spontaneous HFOs when no electric stimulus was applied. DHPG (50 *μ*mol/L), histamine (50 *μ*mol/L) and carbachol (10 *μ*mol/L) slightly changed the power of the stimulus-induced HFOs (DHPG: 98 %; 149 ± 36 %, *P* ≤ 0.0001; *n* = 49) (histamine: 120 %; 127 ± 55 %, *P* ≤ 0.0001; *n* = 38) (carbachol: 104 %; 109 ± 52%, *P* = 0.0081; *n* = 38) (Fig.7A). The addition of glutamate caused a dose-dependent increase in HFO power (10 *μ*mol/L: 133 %; 123 ± 16%; *P* ≤ 0.0001; *n* = 52) (25 *μ*mol/L: 263 %; 198 ± 26 %, *P* ≤ 0.0001; *n* = 52) (50 *μ*mol/L: 667 %; 435 ± 56 %, *P* ≤ 0.0001; *n* = 52) (100 *μ*mol/L: 376 %; 378 ± 44 %; *P* ≤ 0.0001; *n* = 61). All these effects were highly significant. The strong effect of glutamate on HFO power even at low doses is surprising, as block of ionotropic glutamate receptors had no effect and the group I mGluR agonist DHPG only a very small effect on HFOs. Glutamate might cause this strong effect by activation and interaction of several glutamate receptor subtypes.

From these experiments, we conclude that an increase in intracellular Ca^2+^ is necessary but not sufficient for the generation of HFOs. Ca^2+^ entry via voltage-gated channels seems to play a pivotal role while other signaling components – like for instance metabotropic glutamate receptors – might modulate the shape of the elicited HFOs.

### Astrocytic HFOs are blocked by extracellular Ca^2+^

Not only intracellular but also extracellular Ca^2+^ modulates numerous physiological processes. To investigate its impact we varied the CaCl_2_ concentration in the recording solution. For standard control condition we used a HEPES-buffered salt solution with 1 mmol/L CaCl_2_. Higher Ca^2+^ concentrations attenuated HFOs, while lower concentrations strongly augmented them: 3 mmol/L CaCl_2_ in the bath solution suppressed the total power of the oscillations to a median value of 16 % (18 ± 2 %; *P* ≤ 0.0001; *n* = 91) compared to standard conditions and a reduction in the CaCl_2_ concentration to 500 *μ*mol/L tripled HFO power (300 %; 301 ± 31 %; *P* ≤ 0.0001; *n* = 91). The duration und power of HFOs dramatically increased when external Ca^2+^ was reduced to only 200 *μ*mol/L (1650 %; 1271 ± 139 %; *P* ≤ 0.0001; *n* = 91) (Fig.7B). Another aspect was also striking: While the highest median frequency and power of HFOs were measured directly after the stimulus and monotonically decreased afterwards, HFOs in low Ca^2+^ conditions showed a more chaotic pattern with both increasing and decreasing median frequencies and amplitudes over time. Overall, HFOs under Ca^2+^ deficiency seemed to lack the regulation and coordination seen under conditions with higher Ca^2+^ concentrations (Fig.7B).

With even less than 200 *μ*mol/L Ca^2+^ in the extracellular solution, HFOs on some electrodes lasted for several minutes, so that we could not use our standard stimulation paradigm with stimulus pulses every 20 s. Here we noted that only a minority of the electrodes displayed such long oscillations and that the electrodes showing the longest oscillations changed from stimulus to stimulus. This suggests that HFOs are not only locally restricted, monomorphic and inevitable reactions of one independent cell to an electrical stimulus but that there is some kind of plasticity within the astrocyte layer that shapes the characteristics of the response.

### HFOs are slightly affected by blockade of vesicular transmitter release

The strong dependency on intracellular Ca^2+^ and the phasic HFO pattern raised the suspicion that HFOs were a correlate of synaptic-like vesicular transmitter release. Release of great amounts of anionic transmitters-like ATP or glutamate might cause voltage oscillations in the extracellular space. In astrocytes, vesicular release of the transmitters glutamate and ATP has already been demonstrated in numerous studies and was shown to be inhibited by addition of bafilomycin (Araque et al. 2000; Coco et al. 2003). Bafilomycin A1 is a selective inhibitor of the vacuolar-type H^+^-ATPase (V-ATPase), thereby inhibiting the filling of vesicles with transmitters.

**Figure 8:**
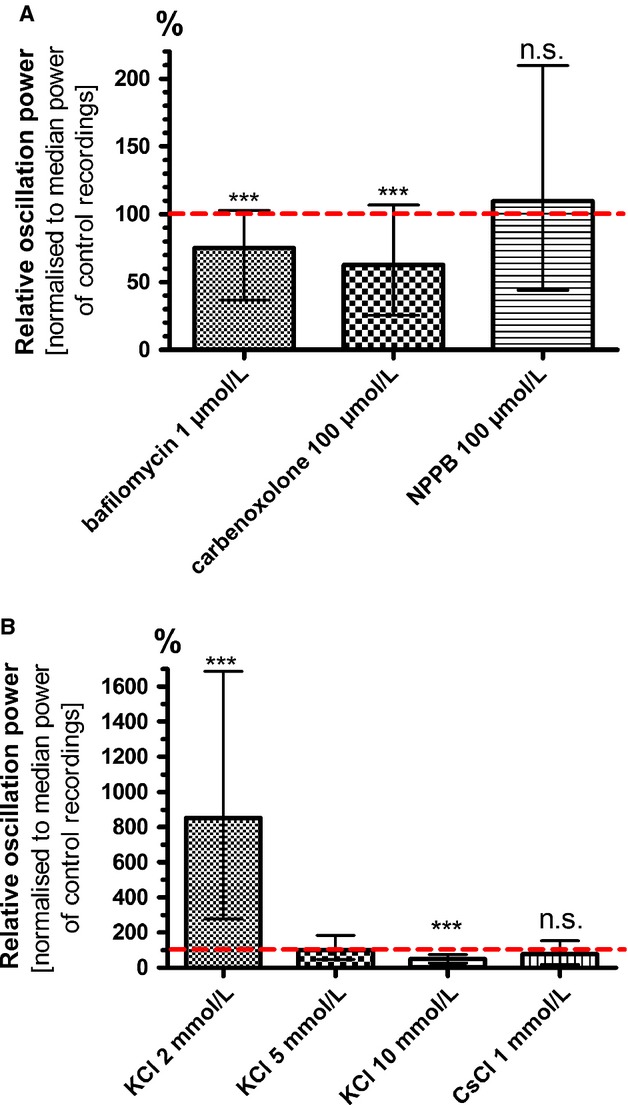
High-frequency oscillations are partially driven by vesicular release of transmitters and are augmented by membrane hyperpolarization. (A) Bafilomycin A1 is an inhibitor of vesicular release. Incubation with 1 *μ*mol/L for 1 h did not completely block but significantly reduced HFO power to 75 % of control recording. A similar effect was seen after application of the gap-junction blocker carbenoxolone (100 *μ*mol/L; 63 %). No inhibition was induced by NPPB (100 *μ*mol/L; 110 %), a blocker of volume-regulated anionic channels. (B) We used various concentrations of KCl in the bath solution to induce changes in the astrocytic membrane potential. Hyperpolarization with 2 mmol/L KCl dramatically raised HFO power to the ninefold of control conditions with 5 mmol/L KCl (870 %). Depolarization with 10 mmol/L KCl on the other hand inhibited HFOs (49 %).The blocker CsCl (1 mmol/L) was applied to standard recording solution to examine whether this effect was due to an involvement of hyperpolarization -activated currents. CsCl had only a slight and not significant effect on HFOs (78 %), suggesting hyperpolarization -activated currents as irrelevant factors in the context of HFOs (***P < 0.001; n.s., not significant).

Incubation with bafilomycin A1 (1 *μ*mol/L for 1 h) had a minor effect on astrocytic HFOs elicited by electrical stimulation: They were not completely blocked, but the oscillation power was reduced to 75 % (65 ± 8 %; *P* ≤ 0.0001; *n* = 59) compared to standard conditions (Fig.8A). This result points to synaptic release as a relevant factor in HFO generation, but not as its sole driving force. Control experiments with neuronal cortical cultures were performed to prove the treatment efficacy: Incubation with bafilomycin led in all cases to a complete inhibition of spontaneous spike activity.

### HFOs are augmented by membrane hyperpolarization

To narrow down factors that contribute to the emergence of HFOs we tested, if the oscillations were susceptible to changes in the membrane potential. Since astrocytes have a big leak conductance for potassium, their membrane potential can be efficiently manipulated by varying the extracellular K^+^ concentration. We tested depolarizing and hyperpolarizing concentrations. Depolarizing the membrane potential with 10 mmol/L KCl in the recording solution instead of 5 mmol/L under control conditions led to a decrease in total oscillation power to 48 % (43 ± 5 %; *P* ≤ 0.0001; *n* = 44). After lowering the extracellular K^+^ concentration from 5 to 2 mmol/L and thereby hyperpolarizing the membrane potential we saw a dramatic increase in oscillation power to 852 % (869 ± 193 %; *P* ≤ 0.0001; *n* = 44; Fig.8B).

This effect might seem counterintuitive because depolarization above a certain threshold level causes neurons to fire action potentials by opening voltage-dependent ion channels. The membrane potential, however, primarily determines the driving force of ions to move into or out of the cell. An enhancement of HFOs by a very negative membrane potential implied that the moving ions have equilibrium potentials at the opposite side of the scale, probably in the high-positive range. This would include Na^+^, Ca^2+^, and intracellular anions like ATP or glutamate. To exclude the possibility that HFOs were triggered by hyperpolarization-activated channels we administered the blocker CsCl in a concentration of 1 mmol/L. Under these conditions, however, HFO power only slightly decreased to 78 % (79 ± 22 %; *P* = 0.0683; *n* = 18; Fig.8B).

### Are hemichannels involved in HFO generation?

One putative way of ions across the membrane is via open hemichannels. Since gap junctions are known to be blocked by Ca^2+^, an involvement of hemichannels in HFO generation would fit our observations of the strong dependency of HFOs to extracellular Ca^2+^. Carbenoxolone is a widely used blocker of gap junctions and hemichannels. Under carbenoxolone (100 *μ*mol/L) treatment, HFOs were significantly reduced in power but not completely abolished 63 % (58 ± 12 %; *P* ≤ 0.0001; *n* = 69; Fig.8A). Thus, hemichannels might play a role in HFO generation by permitting ion flux across the membrane. However, since the specificity of carbenoxolone has recently been questioned, we are advised to interpret these results with caution (Beaumont and Maccaferri 2011). When cortical neuronal cultures were treated with carbenoxolone, spontaneous spike activity nearly vanished. This cannot be explained by a specific action of carbenoxolone, since neuronal network activity is clearly driven by synaptic activity and not by gap junctions.

### Volume-regulated anion channels are not involved in HFO generation

Another way of releasing anions from the cell into the extracellular space is via volume-regulated anion channels (VRACs). Glutamate and ATP were already shown to be released from astrocytes via VRACs (Darby et al. 2003; Anderson et al. 2004; Takano et al. 2005; Kimelberg et al. 2006). VRACs are activated by changes in the cell morphology, for instance by cell swelling, and are permeable for chloride and small organic anions. Astrocytes show fast changes in cell volume and have elaborate mechanisms for its control. Cell volume is not Ca^2+^-dependent *per se*, but increases in intracellular Ca^2+^ have been shown to cause transient astrocyte swelling, thereby activating VRACs. We used 100 *μ*mol/L 5-nitro-2-(3-phenylpropylamino)benzoic acid (NPPB) to block VRACs, but no effect on HFO power of was observed (110 %; 107 ± 13%; *P* = 0.7296; *n* = 57; Fig.8A). Thus, volume-regulated channels are not likely to play a role in HFO generation.

### An increase in extracellular sodium concentration boosts HFOs

**Figure 9:**
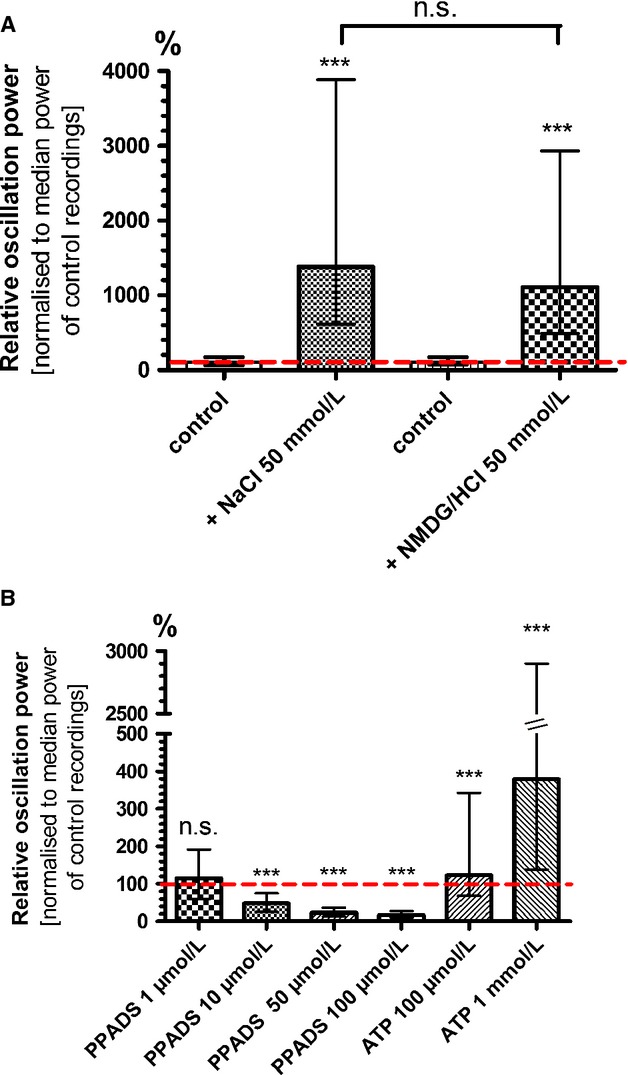
High-frequency oscillations are driven by the movement of – even large – cations into the cells through purinergic receptors. (A) To test our hypotheses that hyperpolarization raised HFO power due to an increased driving force for cations into the cell, we used a bath solution with 150 mmol/L instead of 100 mmol/L NaCl. HFO power dramatically rose to the 14-fold (1377 %) of control conditions. Interestingly, when we added 50 mmol/L NMDG^+^ to the standard bath solution, we saw a comparable rise in HFO power (1106 %). The molecule NMDG^+^ is too big to pass through “normal” ion channels but can enter cells via diluted pore-forming channels. (B) PPADS is a nonselective antagonist of purinergic P2X and P2Y receptors. P2X receptors are ion channels permeable for cations. Here, PPADS dose-dependently decreased HFO power. The natural agonist of these receptors, ATP, increased HFO power only in a rather high concentration of 100 *μ*mol/L (135 % of control recording). We conclude that HFO generation involves P2X receptors with a low affinity for ATP (***P < 0.001; n.s., not significant).

We saw a very strong increase in HFO power when we added extra 50 mmol/L NaCl to the bath solution (150 mmol/L instead of 100 mmol/L NaCl: 1289 %; 1037 ± 218 %; *P* ≤ 0.0001; *n* = 62; Fig.9A). If sodium was indeed the “moving ion” that evoked the HFO, it would be reasonable that the bigger driving force for Na^+^ due to the more positive equilibrium potential with increased Na^+^ concentration would augment HFOs. But by raising the concentration of NaCl we unintentionally shifted the Cl^−^ equilibrium potential as well. However, if Cl^−^ was the “moving ion”, we would have expected increased HFO power after depolarization of the membrane potential. This was not the case. Thus, we conclude that influx of Na^+^ and not of Cl^−^ causes the extracellular voltage oscillations.

For electrophysiological studies Na^+^ can be replaced by the ion *N*-methyl-d-glucamine (NMDG^+^). NMDG^+^ shares some chemical properties with Na^+^, but it is a much bigger molecule and cannot enter the cell via sodium-specific channels. When we added 50 mmol/L NMDG/HCl to the recording solution we expected HFOs to have about the same power as under control conditions. But interestingly, HFOs were boosted to nearly the same level as under 50 mmol/L NaCl extra (1106 %; 944 ± 259 %; *P* ≤ 0.0001; *n* = 46; Fig.9A). Mann–Whitney test revealed no significant difference between the NaCl- and the NMDG/HCl-induced effect on HFO power (*P* = 0.2222).

This might suggest a rather unspecific effect due to the increased osmolarity. But it might also be a hint that the electrical stimulation opens channels in the membrane with pores wide enough to let Na^+^ and big molecules like NMDG^+^ pass in equal measure.

### Indications for the involvement of pore-forming P2X receptors

One proposed mechanism how big molecules like NMDG^+^ enter astrocytes are membrane pores associated with ionotropic purinergic P2X receptors. P2X_2_, P2X_4_, and P2X_7_-receptors have been shown to form pores after several seconds of ATP activation that are permeable to large cations (Jarvis and Khakh 2009). Although the mechanism is not completely understood, it is presumed that either the receptor itself or recruited pannexins form pores that are even wide enough to let fluorescent dyes enter the cell (Suadicani et al. 2006; Hamilton et al. 2008). We used pyridoxalphosphate-6-azophenyl-2′,4′-disulfonic acid tetrasodium salt (PPADS) to block P2 receptors. HFO power dose-dependently decreased when PPADS was admitted to the astrocyte culture (1 *μ*mol/L: 115 %; 109 ± 13%; *P* = 0.2534; *n* = 61) (10 *μ*mol/L: 48 %; 42 ± 5%; *P* ≤ 0.0001; *n* = 61) (50 *μ*mol/L: 23 %; 20 ± 2%; *P* ≤ 0.0001; *n* = 61) (100 *μ*mol/L: 16 %; 15 ± 2 %; *P* ≤ 0.0001; *n* = 61; Fig.9B). These results point to an involvement of P2 receptors, but do not clarify which subtypes are relevant.

PPADS blocks the rat variant of the P2X_4_ receptor inefficiently with an IC_50_ of >100 *μ*mol/L (Jones et al. 2000), the P2X_7_ receptor shows an intermediate sensitivity (53 *μ*mol/L; Watano et al. 2002) and the P2X_2_ receptor the highest sensitivity (~IC_50_ = 6 *μ*mol/L; García-Lecea et al. 1999). These values highlight P2X_7_ receptors as most likely candidates involved in HFO generation as 10 *μ*mol/L PPADS in our experiments already induced a moderate inhibition.

Since pore-forming is thought to be induced by a permanent activation of the receptor with ATP, we elicited HFOs when ATP was applied to the recording solution. We tested increasing concentration of ATP (10 *μ*mol/L, 25 *μ*mol/L, 50 *μ*mol/L, 100 *μ*mol/L; 1 mmol/L), but only the highest concentrations showed a significant effect on HFO power (100 *μ*mol/L: 153%; 150 ± 144%; *P* = 0.0002; *n* = 89; 1 mmol/L: 379%; 1076 ± 1858%; *P* ≤ 0.0001; Fig.9B). These concentrations seem rather high, but in comparison to other P2X receptors, P2X_7_ receptors have a considerably lower affinity to ATP (EC_50_ = 100 *μ*mol/L; Jarvis and Khakh 2009; North 2002; Sun et al. 2013). It is known that low concentrations of divalent cations facilitate the transport through P2X_7_ pores, which very well fits our observations in low-Ca^2+^ solution where HFOs were extremely powerful (Virginio et al. 1997; Nörenberg et al. 2011). P2X_2_ receptors are not blocked by Ca^2+^ and P2X_4_ receptors only at higher concentrations (IC_50_ = 87 *μ*mol/L; Jarvis and Khakh 2009). Thus, the P2X_7_ receptor might be the best candidate for mediating this ion flux that manifests itself as HFOs in extracellular recordings.

### Acidic pH increases the power of HFOs

**Figure 10:**
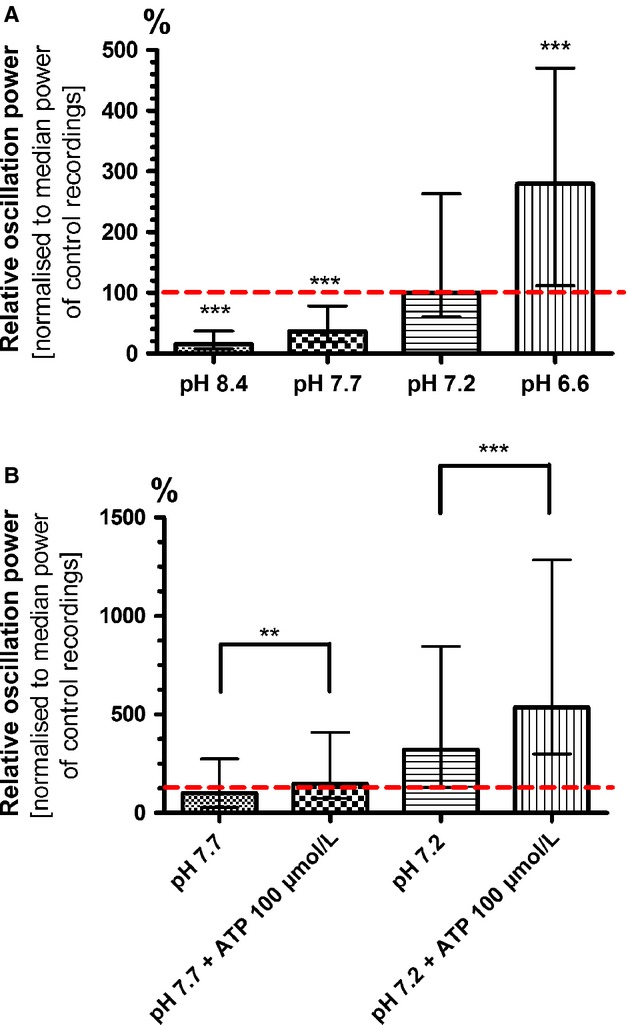
Acidic pH boosts HFOs and sensitizes the cells for ATP. (A) Lowering extracellular pH caused a gradual increase in pH power. Even slight changes in pH (from 7.7 to 7.2) enhanced HFO power threefold. At pH 6.6, HFO power increased threefold compared to 7.2 (279 %) and HFO duration lasted nearly as long as under the calcium deficiency paradigm illustrated in Figure7 B (200 *μ*mol/L calcium). (B) Addition of 100 *μ*mol/L ATP to a recording solution with a pH of 7.7 increased HFO power to 148 %. Incubation with an acidic bath solution of pH 7.2 already tripled HFO power compared to a pH 7.7 control (321 %), but application of ATP further increased power to 536 % (**P < 0.01; ***P < 0.001).

A striking effect on HFOs was seen when the pH of the extracellular solution was changed: Acidification to a minimum pH of 6.6 strongly enhanced HFO power compared to a solution with a pH of 7.2 (279 %; 194 ± 34%; *P* = 0.0007; *n* = 51), while an increased pH (up to 8.4) had the opposite effect (16 %; 19 ± 6 %; *P* ≤ 0.0001; *n* = 51; Fig.10A).

The strengthening effect of ATP (100 *μ*mol/L) on oscillation power was larger at an pH of 7.2 (169 %; 157 ± 33%; *P* ≤ 0.0001; *n* = 43; Fig.10B) compared to the ATP effect at an more basic pH of 7.7 (148%; 117 ± 21%; *P *=* *0.0056; *n* = 43): It seemed as if the low pH had sensitized the astrocytes for the agonist ATP.

## Discussion

### High-frequency oscillations

High-frequency oscillations of field potentials above the gamma band (100—200 Hz) have been recorded in hippocampal and cortical slices but also in the intact brain. These so-called ripples are thought to be field correlates of simultaneous population spike bursts synchronized by recurrent inhibition and have been associated with memory formation during sleep (Buzsáki and Silva 2012). Transient field potential oscillations at even higher frequencies (200–600 Hz) were attributed to pathological conditions, especially in seizure initiation. In a recent study, it was shown that incidence, frequency, and form of the ripples strongly depend on the extracellular calcium concentration. Hippocampal slices bathed in 3 mmol/L Ca^2+^ showed HFOs in the ripple range but turned to fast ripples when the Ca^2+^ was lowered to 1 mmol/L (Aivar et al. 2014). The authors claim that low Ca^2+^ enhances the intrinsic excitability of neurons by shifting the voltage-dependence of Na^+^ channels. This would increase the probability of neuronal bursting, which is a prerequisite for the emergence of fast ripples.

To our knowledge, the direct or indirect involvement of astrocytes in ripple generation has never been discussed. We know that astrocytic Ca^2+^ signaling can lead to neuronal depolarization and spontaneous epileptiform discharges (Kang et al. 2005). On the other hand, low external Ca^2+^ in hippocampal slices efficiently evokes Ca^2+^ oscillations in astrocytes followed by simultaneous Ca^2+^ elevations in adjacent neurons (Fellin et al. 2004). Thus, astrocytes might facilitate neuronal synchrony and epileptiform activity. The lack of a prominent frequency band in astrocytic oscillations described in our study raises doubt whether astrocytes are directly involved in generating ripple-like activity in the brain. Likewise, the observed strong local restriction of HFOs does not support the idea of astrocytes being key players in brain oscillatory activity. However, strong interactions between astrocytes and neurons on several functional levels have already been well established. By giving evidence for extracellular voltage fluctuations produced by astrocytes our study adds new colors to the palette of astrocytic potential to modulate and interfere with neuronal function. Obviously, experiments in mixed cultures will be a necessary next step to address the question how and to which extent these voltage fluctuations affect or are affected by neuronal activity.

### HFOs depend on activation of voltage-gated channels

While the involvement of voltage-gated channels in the emergence of HFOs was clearly shown in our experiments with astrocytes cultures, it still raises some questions: In contrast to neurons, astrocytes are thought to be electrically passive. They show a rather linear voltage/current relationship due to the lack of any voltage-gated ion currents (Adermark and Lovinger 2008). However, the expression of voltage-activated channels has been demonstrated in cultured astrocytes (MacVicar 1984; Latour et al. 2003). Whether this is true for astrocytes in slices or *in situ* still remains under debate. Some authors claim that the presence of voltage-gated channels in non-neuronal brain cells determines a separate class of cells, but a proper nomenclature and classification has not yet been established. These cells are either called complex astrocytes (Adermark and Lovinger 2008), NG2 cells (polydendrocytes; Schools et al. 2003) or OPCs (oligodendrocyte precursor cells; Paez et al. 2009), since they are assumed to be progenitors of oligodendrocytes. OPCs express purinergic receptors in addition to voltage-gated channels, and they respond to glutamate and ATP with a rise of intracellular calcium (Hamilton et al. 2010; Zonouzi et al. 2011).

We cannot make a clear statement whether the cells in our experiments were glial precursor cells or astrocytes that up- or downregulated several genes due to the culturing process. Immunostainings revealed that the vast majority of the cells in our cultures were positive for GFAP (glial fibrillary acidic protein), which is not a feature of NG2 cells in vivo (Nishiyama et al. 2005) but common for astrocytes in vitro.

Strong GFAP expression can be also a sign of reactive astrogliosis – a response to brain trauma, infection, ischemia, or neurodegenerative disorders where astrocytes strongly proliferate and undergo molecular and morphological changes. An upregulation of L-type Ca^2+^channels was reported for astrocytes in several models of brain injury (Westenbroek et al. 1998; Chung et al. 2001; Xu et al. 2007; Willis et al. 2010; Rubio et al. 2013).

### HFO are highly susceptible to changes in extracellular Ca^2+^

Variation in extracellular calcium concentrations affects many signaling compartments, so we cannot directly infer a certain cellular mechanism from the strong calcium dependency. Based on previous studies and observations we can make some assumptions, although. Incubation of hippocampal slices in ACSF with low calcium is an established model of epilepsy. Some researchers claim that in this model seizure-like activity is due to excessive electric coupling, since calcium blocks the transport of molecules through gap junctions (Perez-Velazquez et al. 1994). Uncoupled gap junctions – so called hemichannels – that release or take up substances from the extracellular space are open when external calcium concentrations are low (Quist et al. 2000). Hales et al. (2012) attributed the strong effect of calcium on the observed HFOs in their experiments to the involvement of hemichannels. In addition, low calcium is known to elicit Ca^2+^ oscillations in astrocytes and to enhance the rise in intracellular Ca^2+^ induced by ATP, where it is unclear whether gap junctions are involved (Zanotti and Charles 1997). It was also discussed that low extracellular Ca^2+^ depolarizes the cell membrane possibly by altering the surface charge (Anderson et al. 1995), but membrane depolarization with high extracellular potassium concentrations failed to show the same promoting effect on HFOs as low Ca^2+^ in our experiments. Furthermore, the ion Ba^2+^ which blocks K^+^ channels – thus leading to a depolarization of the cell – did not enhance but significantly weakened HFOs, which nearly vanished when 1 mmol/L BaCl_2_ was added to the bath solution. Hence, it is not very likely that Ca^2+^ influences HFOs by depolarization of the cell membrane.

### HFOs do not depend on synaptic release

Several studies have used bafilomycin A1 to demonstrate vesicular release of gliotransmitters (Araque et al. 2000; Coco et al. 2003; Bowser and Khakh 2007; Gourine et al. 2010): Araque et al. showed that bafilomycin diminished glutamate release while Coco et al. saw the same effect on ATP release. However, in Coco et al. bafilomycin only partially inhibited the gliotransmitter release and it did not affect the initial calcium rise in the stimulated astrocytes. Bowser and Khakh observed that mechanical stimulation in bafilomycin-treated astrocytes induced a calcium rise in some adjacent astrocytes, but strongly suppressed further propagation of the calcium wave. We only saw a minor effect of bafilomycin on the oscillation power. Since previous publications have shown that vesicular release of gliotransmitters is relevant for the propagation of Ca^2+^ waves but not necessarily for their initiation, it is possible that HFOs reflect early steps in a signal cascade that may ultimately lead to propagating Ca^2+^ waves that can spread across a length of up to 200 *μ*m. This explains why HFOs – if they represent early stages of a Ca^2+^ waves – were not detected by any of the neighboring electrodes at a distance of 200 *μ*m.

### HFOs are strengthened by hyperpolarization and increase in extracellular Na^+^

Due to a large resting conductance for potassium, astrocytes have a lower membrane potential than neurons (−85 mV vs. −65 mV; Magistretti and Ransom 2002). Thus, elevation of extracellular K^+^ effectively hyperpolarizes the membrane potential. Assuming an intracellular potassium concentration of 120 mmol/L, lowering the extracellular potassium from 5 to 2 mmol/L K^+^ should – according to the Nernst equation – result in a drift of the membrane potential from approximately −80 to −100 mV. If HFOs were dependent on vesicular release, hyperpolarization of the membrane should rather inhibit HFO power. The observed increase in HFO power after membrane hyperpolarization thus suggests mechanisms other than vesicular release.

Hyperpolarization increases the driving force for ions with equilibrium potentials in the positive range. Thus, influx of Na^+^ and Ca^2+^, as well as efflux of anions like ATP^3−^ and glutamate^-^ are facilitated. Due to the increased driving force for these ions after hyperpolarization, cross-membrane ion currents increase and can contribute to HFOs. On the other hand, K^+^ or Cl^−^ with strongly negative equilibrium potentials are unlikely HFO sources. Possible candidates for channels that could carry such currents are connexin-formed hemichannels, unspecific chloride channels like volume-regulated anionic channels or pore-forming P2X-receptors.

### No indication for an involvement of hemichannels and volume-regulated anionic channels in HFO generation

Hemichannels were discussed as possible sites of glutamate efflux from astrocytes (Parpura et al. 2004; Ye et al. 2009). This transport is markedly blocked by divalent cations such as Ca^2+^, Mg^2+^, and Ba^2+^ in the extracellular solution. Ca^2+^ and Ba^2+^ were also very effective in blocking HFOs in our experiments. Carbenoxolone was used in several studies as blocker of gap junctions and hemichannels that eliminates the electrical coupling between neurons (Zsiros and Maccaferri 2005). Here, it partially inhibited the astrocytic HFO. However, recently the specificity of carbenoxolone was called into question (Beaumont and Maccaferri 2011). The VRAC blocker NPPB had no significant effect on HFO power in our study, giving no indication for VRAC involvement. Incidentally, NPPB was shown to act rather unspecifically as well (Cranmer et al. 1995; Ye et al. 2003).

### Purinergic ATP receptors play a key role in HFO generation

ATP release from astrocytes after electrical stimulation was first described by Guthrie et al. in 1999;. ATP is known to induce autocrine or paracrine ATP release (Anderson et al. 2004), initiating a propagating calcium wave in neighboring astrocytes (Guthrie et al. 1999; Bowser and Khakh 2007) followed by further ATP release. In addition to vesicular and hemichannel-mechanisms, ATP release can occur via pores formed by purinergic P2X receptors (Suadicani et al. 2006; Hamilton et al. 2008). Several subtypes of the ionotropic P2X receptor were shown to form membrane pores after long stimulation with high ATP concentrations that are permeable for larger molecules such as NMDG^+^. We observed comparable increase in HFO power by equimolar amounts of Na^+^ or NMDG^+^, arguing for NMDG^+^ entry into the cell. ATP induces ion currents for instance through P2X_7_ receptors, but they are very small in normal medium containing Ca^2+^ and Mg^2+^. These currents can be greatly increased when the external medium contains a low concentration of these divalent cations (Suadicani et al. 2006; Oliveira et al. 2011). Ca^2+^ is thought to be a negative allosteric modulator of the P2X_7_R by decreasing the receptor's affinity to its ligand ATP (Yan et al. 2011). This behavior fits our observations that low Ca^2+^ caused extremely powerful HFOs. The involvement of purinergic receptors in HFO generation was also demonstrated in our study by the dose-dependent inhibition of HFO power when treated with the unspecific P2 receptor blocker PPADS.

### Acidic pH boosts HFO power

A decrease in pH is an important pathophysiological factor and can be measured in the brain after ischemia, hypoxia, or stroke. HFOs were dramatically strengthened when the pH of the extracellular solution was slightly changed to more acidic values. The opposite effect was seen when we induced HFOs at basic pH. It was already shown in 1997 that low extracellular pH raises intracellular calcium in astrocytes and therefore interacts with astrocytic Ca^2+^ signaling (Nagaoka 1997).

Single channels recordings of P2X_2_ receptor showed that lowering extracellular pH enhanced the affinity for ATP and thereby increased the current through the channel (Ding and Sachs 1999). This fits our data very well, where the effect of ATP (100 *μ*mol/L) on oscillation power grew stronger in an acidic bath solution, while our PPADS measurements rather suggested the P2X_7_ receptor subtype. We therefore propose that different subtypes of purinergic receptors – in combination with other ion channels – contribute to generating HFOs. P2X receptor interplay could also explain HFO strengthening under Ca^2+^ deficiency: P2X_7_ receptors that are blocked by Ca^2+^ show a slower desensitization than other receptors (Jones et al. 2000). Decreasing extracellular Ca^2+^ would then raise the amount of open P2X_7_ receptors with a large conductance that desensitize more slowly than other P2X subtypes that are not inhibited by Ca^2+^.

Our study shows that electrical stimulation triggers fast extracellular voltage oscillations in cultured astrocytes on MEAs. We propose the following mechanism for these astrocytic high-frequency oscillations: The electrical stimulus leads to Ca^2+^ influx through voltage-gated calcium channels, which mediates ATP release and subsequent activation of P2X autoreceptors. Persistent binding at the P2X receptor causes formation of membrane pores that facilitate influx of Na^+^ and Ca^2+^.

## References

[b1] Adermark L, Lovinger DM (2008). Electrophysiological properties and gap junction coupling of striatal astrocytes. Neurochem. Int.

[b2] Aivar P, Valero M, Bellistri E, Menendez de la Prida L (2014). Extracellular calcium controls the expression of two different forms of ripple-like hippocampal oscillations. J. Neurosci.

[b3] Akita T, Fedorovich SV, Okada Y (2011). Ca2+ nanodomain-mediated component of swelling-induced volume-sensitive outwardly rectifying anion current triggered by autocrine action of ATP in mouse astrocytes. Cell. Physiol. Biochem.

[b4] Anderson CM, Bergher JP, Swanson RA (2004). ATP-induced ATP release from astrocytes. J. Neurochem.

[b5] Anderson S, Brismar T, Hansson E (1995). Effect of external K+, Ca2+, and Ba2+ on membrane potential and ionic conductance in rat astrocytes. Cell. Mol. Neurobiol.

[b6] Araque A, Li N, Doyle RT, Haydon PG (2000). SNARE protein-dependent glutamate release from astrocytes. J. Neurosci.

[b7] Araque A, Navarrete M (2010). Glial cells in neuronal network function. Philos. Trans. R. Soc. Lond., B Biol. Sci.

[b8] Barker AJ, Ullian EM (2010). Astrocytes and synaptic plasticity. Neuroscientist.

[b9] Beaumont M, Maccaferri G (2011). Is connexin36 critical for GABAergic hypersynchronization in the hippocampus?. J. Physiol. (Lond.).

[b10] Bennett MV, Barrio LC, Bargiello TA, Spray DC, Hertzberg E, Sáez JC (1991). Gap junctions: new tools, new answers, new questions. Neuron.

[b11] Bowser DN, Khakh BS (2007). Vesicular ATP is the predominant cause of intercellular calcium waves in astrocytes. J. Gen. Physiol.

[b12] Buzsáki G, Silva FLD (2012). High frequency oscillations in the intact brain. Prog. Neurobiol.

[b13] Charles AC, Merrill JE, Dirksen ER, Sanderson MJ (1991). Intercellular signaling in glial cells: calcium waves and oscillations in response to mechanical stimulation and glutamate. Neuron.

[b14] Chung YH, Shin CM, Kim MJ, Cha CI (2001). Enhanced expression of L-type Ca2+ channels in reactive astrocytes after ischemic injury in rats. Neurosci. Lett.

[b15] Coco S, Calegari F, Pravettoni E, Pozzi D, Taverna E, Rosa P (2003). Storage and release of ATP from astrocytes in culture. J. Biol. Chem.

[b16] Cornell-Bell AH, Finkbeiner SM, Cooper MS, Smith SJ (1990). Glutamate induces calcium waves in cultured astrocytes: long-range glial signaling. Science.

[b17] Cranmer SL, Conant AR, Gutteridge WE, Halestrap AP (1995). Characterization of the enhanced transport of L- and D-lactate into human red blood cells infected with Plasmodium falciparum suggests the presence of a novel saturable lactate proton cotransporter. J. Biol. Chem.

[b18] Darby M, Kuzmiski JB, Panenka W, Feighan D, Macvicar BA (2003). ATP released from astrocytes during swelling activates chloride channels. J. Neurophysiol.

[b19] Ding S, Sachs F (1999). Single channel properties of P2X2 purinoceptors. J. Gen. Physiol.

[b20] Fellin T, Pascual O, Gobbo S, Pozzan T, Haydon PG, Carmignoto G (2004). Neuronal synchrony mediated by astrocytic glutamate through activation of extrasynaptic NMDA receptors. Neuron.

[b21] Finkbeiner S (1992). Calcium waves in astrocytes-filling in the gaps. Neuron.

[b22] García-Lecea M, Delicado EG, Miras-Portugal MT, Castro E (1999). P2X2 characteristics of the ATP receptor coupled to [Ca2+]i increases in cultured Purkinje neurons from neonatal rat cerebellum. Neuropharmacology.

[b23] Gourine AV, Kasymov V, Marina N, Tang F, Figueiredo MF, Lane S (2010). Astrocytes control breathing through pH-dependent release of ATP. Science.

[b24] Guthrie PB, Knappenberger J, Segal M, Bennett MV, Charles AC, Kater SB (1999). ATP released from astrocytes mediates glial calcium waves. J. Neurosci.

[b25] Halassa MM, Haydon PG (2010). Integrated brain circuits: astrocytic networks modulate neuronal activity and behavior. Annu. Rev. Physiol.

[b26] Hales CM, Zeller-Townson R, Newman JP, Shoemaker JT, Killian NJ, Potter SM (2012). Stimulus-evoked high frequency oscillations are present in neuronal networks on microelectrode arrays. Front. Neural. Circuits.

[b27] Hamilton N, Vayro S, Kirchhoff F, Verkhratsky A, Robbins J, Gorecki DC (2008). Mechanisms of ATP- and glutamate-mediated calcium signaling in white matter astrocytes. Glia.

[b28] Hamilton N, Vayro S, Wigley R, Butt AM (2010). Axons and astrocytes release ATP and glutamate to evoke calcium signals in NG2-glia. Glia.

[b29] Hassinger TD, Guthrie PB, Atkinson PB, Bennett MV, Kater SB (1996). An extracellular signaling component in propagation of astrocytic calcium waves. Proc. Natl Acad. Sci. USA.

[b30] Jarvis MF, Khakh BS (2009). ATP-gated P2X cation-channels. Neuropharmacology.

[b31] Jeremic A, Jeftinija K, Stevanovic J, Glavaski A, Jeftinija S (2001). ATP stimulates calcium-dependent glutamate release from cultured astrocytes. J. Neurochem.

[b32] Jones CA, Chessell IP, Simon J, Barnard EA, Miller KJ, Michel AD (2000). Functional characterization of the P2X(4) receptor orthologues. Br. J. Pharmacol.

[b33] Kang N, Xu J, Xu Q, Nedergaard M, Kang J (2005). Astrocytic glutamate release-induced transient depolarization and epileptiform discharges in hippocampal CA1 pyramidal neurons. J. Neurophysiol.

[b34] Kimelberg HK, Macvicar BA, Sontheimer H (2006). Anion channels in astrocytes: biophysics, pharmacology, and function. Glia.

[b35] Latour I, Hamid J, Beedle AM, Zamponi GW, Macvicar BA (2003). Expression of voltage-gated Ca2+ channel subtypes in cultured astrocytes. Glia.

[b36] Liu H, Akita T, Shimizu T, Sabirov RZ, Okada Y (2009). Bradykinin-induced astrocyte-neuron signalling: glutamate release is mediated by ROS-activated volume-sensitive outwardly rectifying anion channels. J. Physiol. (Lond.).

[b37] MacVicar BA (1984). Voltage-dependent calcium channels in glial cells. Science.

[b38] Magistretti PJ, Nemeroff C, Ransom BR, Davis KL, Charney D, Coyle JT (2002). Astrocytes. Neuropsychopharmacology. The fifth generation of progress; an official publication of the American College of Neuropsychopharmacology.

[b39] Nagaoka TMATT (1997). Lowering extracellular pH raises intracellular calcium in cultured rat astrocytes. Acta Histochem. Cytochem.

[b40] Nase G, Helm PJ, Enger R, Ottersen OP (2008). Water entry into astrocytes during brain edema formation. Glia.

[b41] Nishiyama A, Yang Z, Butt A (2005). Astrocytes and NG2-glia: what's in a name?. J. Anat.

[b42] Nörenberg W, Hempel C, Urban N, Sobottka H, Illes P, Schaefer M (2011). Clemastine potentiates the human P2X7 receptor by sensitizing it to lower ATP concentrations. J. Biol. Chem.

[b43] North RA (2002). Molecular physiology of P2X receptors. Physiol. Rev.

[b44] Oliveira JF, Riedel T, Leichsenring A, Heine C, Franke H, Krügel U (2011). Rodent cortical astroglia express in situ functional P2X7 receptors sensing pathologically high ATP concentrations. Cereb. Cortex.

[b45] Oostenveld R, Fries P, Maris E, Schoffelen J (2011). FieldTrip: Open source software for advanced analysis of MEG, EEG, and invasive electrophysiological data. Comput. Intell. Neurosci.

[b46] Ota Y, Zanetti AT, Hallock RM (2013). The role of astrocytes in the regulation of synaptic plasticity and memory formation. Neural. Plast.

[b47] Paez PM, Fulton D, Colwell CS, Campagnoni AT (2009). Voltage-operated Ca(2+) and Na(+) channels in the oligodendrocyte lineage. J. Neurosci. Res.

[b48] Pangrsic T, Potokar M, Haydon PG, Zorec R, Kreft M (2006). Astrocyte swelling leads to membrane unfolding, not membrane insertion. J. Neurochem.

[b49] Parpura V, Basarsky TA, Liu F, Jeftinija K, Jeftinija S, Haydon PG (1994). Glutamate-mediated astrocyte-neuron signalling. Nature.

[b50] Parpura V, Scemes E, Spray DC (2004). Mechanisms of glutamate release from astrocytes: gap junction “hemichannels”, purinergic receptors and exocytotic release. Neurochem. Int.

[b51] Pasti L, Volterra A, Pozzan T, Carmignoto G (1997). Intracellular calcium oscillations in astrocytes: a highly plastic, bidirectional form of communication between neurons and astrocytes in situ. J. Neurosci.

[b52] Perez-Velazquez JL, Valiante TA, Carlen PL (1994). Modulation of gap junctional mechanisms during calcium-free induced field burst activity: a possible role for electrotonic coupling in epileptogenesis. J. Neurosci.

[b53] Quist AP, Rhee SK, Lin H, Lal R (2000). Physiological role of gap-junctional hemichannels. Extracellular calcium-dependent isosmotic volume regulation. J. Cell Biol.

[b54] Reetz G, Wiesinger H, Reiser G (1997). ATP-induced oscillations of cytosolic Ca2+ activity in cultured astrocytes from rat brain are modulated by medium osmolarity indicating a control of [Ca2+]i oscillations by cell volume. Neurochem. Res.

[b55] Rubio N, Almanza A, Mercado F, Arévalo MÁ, Garcia-Segura LM, Vega R (2013). Upregulation of voltage-gated Ca2+ channels in mouse astrocytes infected with Theiler's murine encephalomyelitis virus (TMEV). Neuroscience.

[b56] Santello M, Calì C, Bezzi P (2012). Gliotransmission and the tripartite synapse. Adv. Exp. Med. Biol.

[b57] Schools GP, Zhou M, Kimelberg HK (2003). Electrophysiologically “complex” glial cells freshly isolated from the hippocampus are immunopositive for the chondroitin sulfate proteoglycan NG2. J. Neurosci. Res.

[b58] Stout CE, Costantin JL, Naus CCG, Charles AC (2002). Intercellular calcium signaling in astrocytes via ATP release through connexin hemichannels. J. Biol. Chem.

[b59] Suadicani SO, Brosnan CF, Scemes E (2006). P2X7 receptors mediate ATP release and amplification of astrocytic intercellular Ca2+ signaling. J. Neurosci.

[b60] Sun C, Heid ME, Keyel PA, Salter RD (2013). The second transmembrane domain of P2X7 contributes to dilated pore formation. PLoS One.

[b61] Takano T, Kang J, Jaiswal JK, Simon SM, Lin JH, Yu Y (2005). Receptor-mediated glutamate release from volume sensitive channels in astrocytes. Proc. Natl Acad. Sci. USA.

[b62] Thrane AS, Rappold PM, Fujita T, Torres A, Bekar LK, Takano T (2011). Critical role of aquaporin-4 (AQP4) in astrocytic Ca2+ signaling events elicited by cerebral edema. Proc. Natl Acad. Sci. USA.

[b63] Virginio C, Church D, North RA, Surprenant A (1997). Effects of divalent cations, protons and calmidazolium at the rat P2X7 receptor. Neuropharmacology.

[b64] Watano T, Matsuoka I, Kimura J (2002). Characteristics of ATP-induced current through P2X7 receptor in NG108-15 cells: unique antagonist sensitivity and lack of pore formation. Jpn. J. Pharmacol.

[b65] Westenbroek RE, Bausch SB, Lin RC, Franck JE, Noebels JL, Catterall WA (1998). Upregulation of L-type Ca2+ channels in reactive astrocytes after brain injury, hypomyelination, and ischemia. J. Neurosci.

[b66] Willis M, Kaufmann WA, Wietzorrek G, Hutter-Paier B, Moosmang S, Humpel C (2010). L-type calcium channel CaV 1.2 in transgenic mice overexpressing human AbetaPP751 with the London (V717I) and Swedish (K670M/N671L) mutations. J. Alzheimers Dis.

[b67] Xu JH, Long L, Tang YC, Hu HT, Tang FR (2007). Ca(v)1.2, Ca(v)1.3, and Ca(v)2.1 in the mouse hippocampus during and after pilocarpine-induced status epilepticus. Hippocampus.

[b68] Yan Z, Khadra A, Sherman A, Stojilkovic SS (2011). Calcium-dependent block of P2X7 receptor channel function is allosteric. J. Gen. Physiol.

[b69] Yatani A, Seidel CL, Allen J, Brown AM (1987). Whole-cell and single-channel calcium currents of isolated smooth muscle cells from saphenous vein. Circ. Res.

[b70] Ye Z, Oberheim N, Kettenmann H, Ransom BR (2009). Pharmacological “cross-inhibition” of connexin hemichannels and swelling activated anion channels. Glia.

[b71] Ye Z, Wyeth MS, Baltan-Tekkok S, Ransom BR (2003). Functional hemichannels in astrocytes: a novel mechanism of glutamate release. J. Neurosci.

[b72] Zanotti S, Charles A (1997). Extracellular calcium sensing by glial cells: low extracellular calcium induces intracellular calcium release and intercellular signaling. J. Neurochem.

[b73] Zonouzi M, Renzi M, Farrant M, Cull-Candy SG (2011). Bidirectional plasticity of calcium-permeable AMPA receptors in oligodendrocyte lineage cells. Nat. Neurosci.

[b74] Zorec R, Araque A, Carmignoto G, Haydon PG, Verkhratsky A, Parpura V (2012). Astroglial excitability and gliotransmission: an appraisal of Ca2+ as a signalling route. ASN Neuro.

[b75] Zsiros V, Maccaferri G (2005). Electrical coupling between interneurons with different excitable properties in the stratum lacunosum-moleculare of the juvenile CA1 rat hippocampus. J. Neurosci.

